# The Relationship Between Choroidal and Photoreceptor Layer Thickness With Visual Acuity in Highly Myopic Eyes

**DOI:** 10.3389/fncel.2022.800065

**Published:** 2022-02-02

**Authors:** Mazaya Mahmud, Amin Ahem, Mae-Lynn Catherine Bastion, Rokiah Omar, Azmawati Mohammed Nawi, Norsyariza Razak, Adib Mohd Satali, Safinaz Mohd Khialdin, Norshamsiah Md Din

**Affiliations:** ^1^Department of Ophthalmology, Faculty of Medicine, Universiti Kebangsaan Malaysia Medical Centre, Kuala Lumpur, Malaysia; ^2^Department of Ophthalmology, Faculty of Medicine, Universiti Putra Malaysia, Seri Kembangan, Malaysia; ^3^Optometry and Vision Science Programme, Faculty of Health Science, School of Healthcare Sciences, Universiti Kebangsaan Malaysia, Kuala Lumpur, Malaysia; ^4^Department of Public Health, Universiti Kebangsaan Malaysia Medical Centre, Kuala Lumpur, Malaysia

**Keywords:** high myopia, choroidal thickness, photoreceptor thickness, visual acuity, optical coherence tomography

## Abstract

**Purpose:**

The aim of this study was to evaluate the choroidal and photoreceptor thickness in highly myopic eyes and its correlation with visual acuity.

**Methods:**

This is a cross-sectional, observational study involving 57 eyes of 57 highly myopic subjects [spherical equivalent ≥ –6 diopters (D) or axial length ≥ 26 mm] seen in a tertiary institutional center. Eyes with any clinical evidence of maculopathy or amblyopia were excluded. All subjects underwent a refraction assessment, visual acuity, axial length measurement using the IOL Master, and full ocular assessment. Eyes were imaged using Spectralis Optical Coherence Tomography by one experienced operator. Two independent investigators manually measured subfoveal choroidal thickness (SFCT) and foveal photoreceptor thickness (FPT).

**Results:**

The mean SFCT was 195.88 ± 87.63 μm (range: 32–373) and mean FPT was 96.68 ± 11.23 μm (range: 67–100), after correction for ocular magnification. The best corrected visual acuity (BCVA) in LogMAR was negatively correlated with SFCT (*r* = –0.510, *p* = 0.001) and FPT (*r* = –0.397, *p* = 0.002) and positively correlated with age (*r* = 0.418, *p* = 0.001) and axial length (*r* = 0.551, *p* = 0.001). Multiple linear regression analysis showed that age, axial length, and corrected FPT were significant risk factors for poorer BCVA (*p* = 0.021, < 0.001, and 0.02, respectively).

**Conclusion:**

FPT, age, and axial length are significant moderate predictive factors for poorer visual acuity in highly myopic eyes without myopic maculopathy. Thinner SFCT does not translate into poorer vision.

## Introduction

Myopia is a common condition affecting a significant proportion of the population particularly in East Asian countries (Saw et al., 2005; Luo et al., 2006). It is classified into low, moderate, and high myopia based on the spherical refractive power (SRP). Low myopia describes SRP from –0.25 to –3.00 diopters (D), moderate myopia between –3.00 and –6.00 D, high myopia is SRP of –6.00 diopters (D) or greater, and an axial length exceeding 26 mm (Curtin, 1979). In highly myopic eyes, excessive globe elongation can cause mechanical stretching and thinning of the choroid and retinal pigment epithelium (RPE) layers, resulting in various retinal degenerative changes such as peripheral retinal degenerations, retinal tears, retinal detachments, posterior staphyloma, chorioretinal atrophy, RPE dystrophy, macular holes, lacquer cracks, choroidal neovascularization, and macula hemorrhage (Soubrane, 2008; Ikuno, 2017). High myopia associated with retinal complications is also known as pathological myopia.

The rate of high myopia and possibly pathological myopia appears to be rising in Asia and other parts of the world. The apparent worldwide rise in the prevalence of myopia has a large public impact due to the associated increase in potentially blinding ocular complications. Decreased visual acuity in the presence of maculopathy is explainable. However, the pathological mechanisms for decreased visual acuity in the absence of observable macular changes have not been clarified.

Optical coherence tomography (OCT) is a non-contact, non-invasive technique to measure the retinal thickness and optic discs of healthy and diseased eyes. Spectral-domain OCT (SD-OCT) provides higher resolution images of the posterior fundus with faster scanning speed and image processing (Spaide et al., 2008; Wong et al., 2011). The enhanced depth imaging mode on the OCT (EDI-OCT) is able to image deeper structures in the eye including the choroid to assess structural alterations induced by high myopia (Spaide et al., 2008). EDI mode moves the imaging focal point of SD-OCT (the zero-delay line) more posteriorly, allowing a stronger returning reflection from the choroid, resulting in a better definition of details (Mrejen and Spaide, 2013).

We aimed to determine the relationship between visual acuity and both foveal photoreceptor and choroidal thickness in highly myopic eyes to better explain the possible causes of decreased visual acuity in the absence of macular pathology in this group of patients.

## Materials and Methods

This is a cross-sectional study involving patients who attended the Ophthalmology and Optometry clinic from June 2014 to June 2015. The inclusion criteria were individuals aged between 18 and 80 years old with spherical equivalent (SE) of –6.0 D or greater in at least one eye. The exclusion criteria were underlying ocular comorbidities such as glaucoma, uveitis or retinal detachment, any previous ocular procedure including cataract surgery, refractive surgery, vitreoretinal surgery or laser procedure, amblyopia, eyes with axial length <26 mm (to exclude lenticular myopia with cataract), eyes with corneal abnormality such as keratoconus or corneal scars, presence of dense cataract preventing detailed fundus examination and good OCT signal strength, and eyes with macular abnormalities attributed to pathological myopic changes (e.g., lacquer cracks in the fovea, choroidal neovascularization, and myopic macular schisis), or not attributed to pathological myopic changes (e.g., diabetic macular oedema and epiretinal membrane). While it is impossible to exclude undiagnosed or unrecognized amblyopia, we excluded established amblyopia such as refractive or anisometropic amblyopia to eliminate amblyopia as the confounding factor in this study. Additionally, we took the better of two eyes from each subject. Detailed history prior to study recruitment also helped to exclude the history of amblyopia from young. The Research Governance Committee of UKM Medical Center approved the data collection (protocol FF-2014-332). The study adhered to the tenets of the Declaration of Helsinki. Informed consent was taken from all subjects.

Refraction and best-corrected visual acuity (BCVA) were performed by a trained optometrist using the Early Treatment Diabetic Retinopathy Study (ETDRS) charts at 4 m. The axial length measurement was obtained using partial optical coherence interferometry (IOL-Master; Carl Zeiss Meditec Inc.). Detailed ocular examination including fundus examination was performed by the principal investigator.

The EDI-SD-OCT was performed by a single experienced operator using The Spectralis SD-OCT (Heidelberg Engineering, Heidelberg, Germany). The identity of the subject was recorded as numerical code to mask the investigators who interpreted the scan. Pupils were pharmacologically dilated prior to OCT examination. The macula was scanned using 100 averaged scans obtained using eye-tracking within a 5 × 30° rectangle encompassing the center of the fovea horizontally. Only OCT with a good signal strength of >25 was included, as recommended by the manufacturer.

### Measurement of Choroidal and Photoreceptor Layer

**FIGURE 1:**
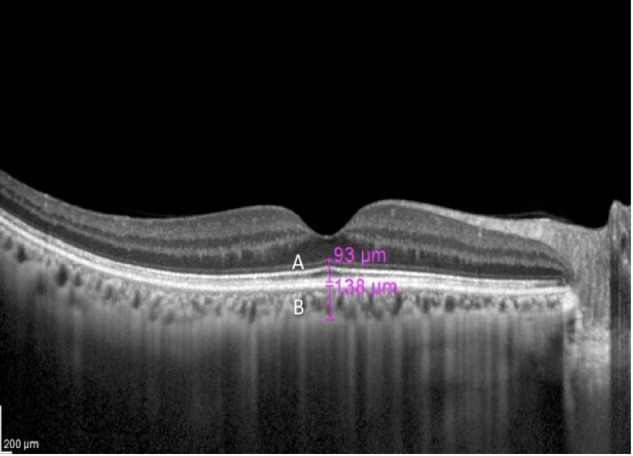
A macula optical coherence tomography (OCT) section of a 67-year-old with myopia of –6.5 D. The magenta lines indicate foveal photoreceptor thickness (FPT) **(A)** and subfoveal choroidal thickness (SFCT) **(B)**.

The foveal photoreceptor thickness (FPT) was defined as the vertical distance between the external limiting membrane (ELM) and the inner border of the RPE (Figure 1A) measured at the lowest foveal depression (Curcio et al., 2000; Ozkaya et al., 2017). The inner segment/outer segment (IS/OS) junction, encompassed in the ELM-RPE layer, has been shown to be the first structure to be affected by any pathology or disease such as retinal detachment (Sakai et al., 2003), retinitis pigmentosa (Milam et al., 1996), and macula hole (Bottoni et al., 2011), and therefore, is predictive for visual acuity. It is also the first structure to recover following treatment (Bottoni et al., 2011).

The subfoveal choroidal thickness (SFCT) was defined as the vertical distance from the outer border of the RPE to the inner border of the sclera in the same area (Figure 1B). Measurements were performed manually using calipers provided by the software by two examiners independently (MM and AA). If the difference in the measurements of the two examiners was >10%, then there will be open adjudication and agreement between the two examiners. If the discrepancy is <10%, the average of the two readings was used.

The FPT and SFCT measurements were corrected for ocular magnification using the Bennett’s formula, *t* = *p* × *q* × *s* (*t* as the real scan length, *p* as the magnification factor determined by the OCT imaging system camera, *q* as the magnification factor related to the eye, and *s* as the original measurement from the OCT image), to adjust the image magnification based on the AL (Bennett et al., 1994). The correction factor *q* was determined using the formula *q* = 0.01306 × (AL - 1.82). Since all of our eyes had AXL of more than 24.46 mm, the real scan length *t* was determined by the equation *t* = (AL - 1.82)/22.64 × *s* (Ye et al., 2019). We also minimized the potential artifact related to magnification or minification due to varying axial length by referring to the scale at the bottom left in all OCT images to standardize the measurements of the OCT parameters. Furthermore, we accepted only OCT images when the fovea is located at the center of the scanning square. While sloppy maculae can be seen in some high myopic subjects, measurements of the FPT and SFCT are made with callipers placed perpendicular to the respective retinal layers to minimize this artifact.

### Statistical Analysis

All continuous data were analyzed using an unpaired *t*-test when comparing the mean between two groups of gender and ethnicity and two categorized groups of axial length. One-way ANOVA was used to compare means of OCT variables between three age groups (<40 years, 40–60 years, and >60 years of age), Pearson correlation was used to examine the correlation between BCVA, age, and axial length with the OCT parameters. Multiple linear regression was used to evaluate the factors affecting the OCT variables and BCVA. Statistical analysis was performed using Statistical Package for Social Science (SPSS) version 22.0. A *p*-value < 0.05 was accepted as statistically significant.

## Results

### Demographic Data

A total of 57 highly myopic eyes from 57 subjects were included in this study. The mean age was 42.4 ± 17.42 years (range 18–72). Slightly more than half of them (56.1%) were <40 years old followed by >60 years old (26.3%) and 40–60 years old (17.5%). There was a slight female predilection with the male to female ratio of 1:1.3. Malays comprised slightly more than half of the population (52.6%), followed by Chinese (40.4%), Indian (3.5%), and other ethnicities (3.5%, 1 native and 1 Portuguese Eurasian descent).

The median SE was –8.25 D (interquartile range, IQR = 2.50, range: –6.25 to –23.00 D). Median axial length was 27.03 mm (IQR = 1.40, range: 26.0–31.4 mm) and median LogMAR BCVA was 0.08 (IQR = 0.20, range: 0–0.52).

The difference between corrected and uncorrected SFCT and FPT was statistically significant with uncorrected mean SFCT of 175.44 ± 81.55 μm (range: 32–373) and corrected mean SFCT of 195.88 ± 87.63 μm. The uncorrected mean FPT was 85.65 ± 10.06 μm (range: 67–100 μm) and the corrected mean FPT was 96.68 ± 11.23 μm (Table 1).

**TABLE 1:** The optical coherence tomography (OCT) measurements with and without correction for ocular magnification.

OCT parameter	Uncorrected measurement	Corrected measurement	Mean difference (95% CI)	*P*-value
SFCT, μm	175.44 ± 81.55	195.88 ± 87.63	20.44 (22.76, 18.11)	<0.001*
FPT, μm	85.65 ± 10.06	96.68 ± 11.22	11.03 (12.27, 9.79)	<0.001*

*Paired t-test.

### Association Between Subfoveal Choroidal Thickness and Foveal Photoreceptor Thickness With Study Variables

One-way ANOVA found a significant difference in corrected SFCT (cSFCT) between the three age groups, and a *post-hoc* Bonferroni found that the different lies between subjects aged <40 years and >60 years (mean difference = 69.91; CI: 3.21, 125.83; *p* = 0.037). cSFCT was also significantly thinner in eyes with axial length ≥ 28 mm μm (121.95 ± 58.64) compared with eyes with axial length <28 mm (213.55 ± 84.48 μm, *p* = 0.001). However, there was no significant difference in corrected FPT (cFPT) between different categorized age groups and axial lengths. There was also no significant difference in cFPT and cSFCT between gender and ethnicity.

**FIGURE 2:**
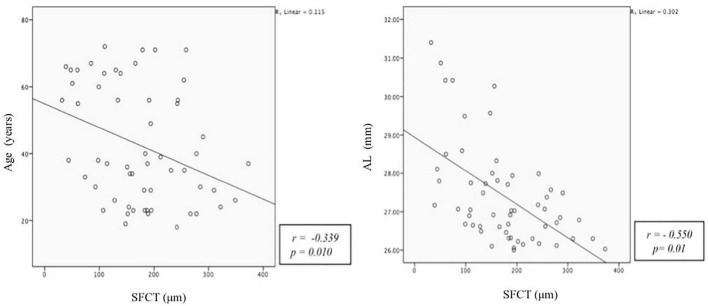
Scatter plot demonstrating the correlation between subfoveal choroidal thickness (SFCT, mm) with age (years) and axial length (AXL, mm). The Y-axis does not begin at zero.

Pearson correlation showed that thinner cSFCT was weakly associated with advancing age (*r* = –0.354, *p* = 0.01) and moderately associated with longer axial length (*r* = –0.475, *p* = 0.0002). No significant correlation was found between cFPT with axial length, age, and BCVA, Table 2). Figure 2 shows a scatter plot of the association between cSFCT with age and axial length.

**TABLE 2:** Association of corrected subfoveal choroidal thickness (SFCT) with age and axial length.

Variable	Regression coefficient (95% CI)	*p*-value*
Age	–2.75 (–2.69, –0.42)	<0.001
Axial length	–29.65 (–44.75, –14.55	0.008

*Multiple linear regression.

A multivariate linear regression was modeled to evaluate factors affecting cSCFT. We included both age and axial length in the model. We found both age and axial length to be significant risk factors for thinner cSCFT. With every 1-year increase in age, cSFCT decline by 1.36 μm, and with every 1 mm increase in axial length, cSCFT decline by 32.84 μm (Table 2). Similar modeling for FPT did not show a significant association with age and axial length.

### Correlation Between Best Corrected Visual Acuity and Study Variables

**FIGURE 3:**
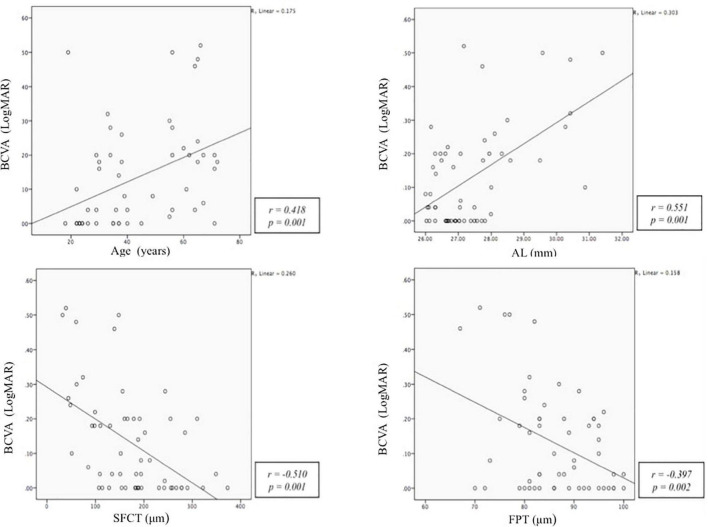
Scatter plots of the best-corrected visual acuity (BCVA, LogMAR) with age (years), axial length (AXL, mm), subfoveal choroidal thickness (SFCT, μm), and foveal photoreceptor thickness (FPT, μm).

Pearson correlation analysis was performed between BCVA, cSFCT, cFPT, age, and axial length (Table 3). We found BCVA to be significantly correlated with all parameters, with moderate correlation with axial length (*r* = 0.551, *p* = 0.001) and cSFCT (*r* = –0.510, *p* = 0.001) and weak correlation with age (*r* = 0.418, *p* = 0.001) and cFPT (*r* = –0.397, *p* = 0.002). Advancing age, longer axial length, and thinner cSFCT and cFPT are significantly correlated with poorer BCVA. Significant correlations of BCVA with study parameters are shown in Figure 3.

**TABLE 3:** Pearson correlation between study variables.

	BCVA	Age	AL	cSFCT
Age	*r* = 0.418** ***p* = 0.001**			
AXL	*r* = 0.551** ***p* = 0.001**	*r* = 0.100 *p* = 0.458		
cSFCT	*r* = –0.510** ***p* = 0.001**	*r* = –0.339** ***p* = 0.010**	*r* = –0.550** ***p* = 0.001**	
cFPT	*r* = –0.397 ** ***p* = 0.002**	*r* = –0.270* ***p* = 0.042**	*r* = **-**0.240 *p* = 0.73	*r* = 0.066 *p* = 0.624

**Statistically significant correlation < 0.01.

*Statistically significant correlation < 0.05.

*BCVA, best corrected visual acuity; AL, axial length; cSFCT, corrected subfoveal choroidal thickness; cFPT, corrected foveal photoreceptor thickness. r = is the correlation coefficient. p = is the statistical significance value from the pearson correlation analysis. The bold values are those which are statistically significant.*

Multiple linear regression analysis was performed to evaluate the association among BCVA, age, axial length, cSFCT, and cFPT to look for the factors that influence BCVA the most (Table 4). We included age, axial length, cSFCT, and cFPT as independent variables and BCVA as the dependent variable in the model. After adjusting for cSFCT, age, axial length, and cFPT were significantly associated with poorer BCVA (*p* = 0.021, <0.001, and 0.02, respectively). The LogMAR BCVA will increase by 0.02 log unit with every 10-year increase in age, increase by 0.05 log unit with every 1 mm increase in axial length, and increase by 0.03 log unit with every 10 μm reduction in cFPT.

**TABLE 4:** Association between BCVA with age, axial length, corrected SFCT, and corrected FPT.

Variable	Regression coefficient (95% CI)	*p*-value*
Age	**0.002 (0.0–0.004)**	**0.021**
Axial length	**0.05 (0.03–0.079)**	**<0.001**
cSFCT	–0.0003 (–0.0007, 0.00005)	0.083
cFPT	-**0.0032 (**-**0.006,** -**0.0005)**	**0.020**

*Multiple linear regression.

*BCVA, best-corrected visual acuity; cSFCT, corrected subfoveal choroidal thickness; cFPT, corrected foveal photoreceptor thickness. The bold values are statistically significant values.*

## Discussion

Highly myopic eyes not only have thinner retina (Lam et al., 2007; Cheng et al., 2010; Hwang and Kim, 2012) but also thinner choroid (Ikuno and Tano, 2009; Chen et al., 2012; Nishida et al., 2012). This structural thinning was recently found to be linked with reduced retinal function (Wolsley et al., 2008). We aimed to see whether this is, in fact, the case in highly myopic eyes in our local Asian population and whether it is the reduction in photoreceptor thickness as the cause of the poor vision. Understanding the mechanism for suboptimal vision in highly myopic eyes without observable myopic maculopathy is important as it would help in understanding the structure-function relationship of retinal degeneration in high myopia.

We defined FPT as the distance between the ELM and inner border of the RPE layer because the IS/OS layer is encompassed in this layer. The IS/OS junction has been shown to be the first structure to be affected in degenerative conditions, followed by damage of the photoreceptor cell bodies occurring later in the process, and the first to recover following treatment (Milam et al., 1996; Sakai et al., 2003; Bottoni et al., 2011).

We acknowledged that the potential artifact ocular magnification may have on the SFCT and FPT thickness measurement in our study. In a study among low myopes between ± 3D, while the authors found a significant difference for lateral measurements (i.e., foveal slope and diameter) between corrected and uncorrected measurements, no significant difference was found between corrected and uncorrected measurements for foveal depth (Parthasarathy and Bhende, 2015). While some studies on high myopes utilized ocular magnification correction (Ye et al., 2019), other studies on high myopes (Luo et al., 2006; Wu et al., 2008; Chung et al., 2019) did not utilize ocular magnification correction, indicating the discrepancies in their use. Additionally, Odell et al., 2011 suggested that any aberrant error is particularly relevant when comparing with macular thickness maps to the normative database or measuring volumes or areas, which is not the case in our study. We found a significant difference between corrected and uncorrected measurements indicating the larger effect that ocular magnification has on high myopic eyes.

Our study found comparable mean thickness in cSFCT (174.84 ± 82.74 μm) with other studies performed on highly myopic eyes among Asians, although it is thicker when compared with a Caucasian population (Nishida et al., 2012; Flores-Moreno et al., 2013). Variations in SFCT were found in different populations and ethnicities in myopic eyes of >6 D, with higher readings among Asians (172.9 ± 72.8 μm) and lower readings among Caucasians (113.3 ± 53.9) (Nishida et al., 2012). However, in the presence of posterior staphyloma, the choroidal thickness can be even thinner even in Asians (100.5 ± 56.9 μm) (Ikuno and Tano, 2009). These disparities demonstrate not only variations among different ethnicities but are also affected by structural abnormalities. All these studies involved high myopic eyes with the normal macula. Our local population, comprising Malay, Chinese, and Indian ethnicities had mean SFCT comparable with other Asian data. However, these readings are far thinner than that in normal (non-myopic) population, varying between 342 ± 118 μm and 354 ± 111 μm (Ikuno et al., 2010; Li et al., 2011).

### Factors Affecting Subfoveal Choroidal Thickness

Our study showed a significant decline in cSFCT with increasing age, with the thinnest among those aged >60 years old, conforming to previous reports among highly myopic eyes of participants with an age range between 49 and 50 years old (Wang et al., 2015; Teberik and Kaya, 2017). The decrease in SFCT is reported between 14 and 15.6 μm with every 10-year increase in age in normal eyes (Margolis and Spaide, 2009; Ikuno et al., 2010). However, our study may suggest that the decline in SFCT can be accelerated in high myopes at 20.75 μm with every 10-year increase in age among our cohort of patients. Apart from microvascular loss with aging (Ding et al., 2011), elongations of the globe may contribute to this rapid decline in high myopes.

We found cSFCT to be significantly thinner in axial length more than 28 mm with a significant negative correlation with axial length, also conforming to previous reports on eyes with high myopia (Nishida et al., 2012; Flores-Moreno et al., 2013). It was postulated that reduction in choroidal thickness is related to excessive globe expansion in longer axial length (Ikuno and Tano, 2009; Chen et al., 2012; Nishida et al., 2012).

We found that the mean cSFCT was not statistically different between genders and ethnicities. The unequal distribution of ethnicities with Malay and Chinese making up the majority may contribute to this result. However, Bafiq et al. (2015) also reported no significant difference in SFCT in blacks, whites, and south Asian origin. We proposed a study with a larger sample size to look into gender and ethnicity factors with choroidal thickness.

### Factors Affecting Foveal Photoreceptor Thickness

Our mean cFPT was 96.68 ± 11.22 μm, slightly higher than a report by Kim et al. (2020) in high myopic eyes (uncorrected subfoveal photoreceptor layer of 72.86 ± 3.35 μm). They found no significant difference in foveal photoreceptor layer between high myopic and normal eyes, and no significant difference between mean FPT and gender (Kim et al., 2020), agreeing with us.

Interestingly, we found cFPT to correlate with BCVA and advancing age but not with longer axial length and thinner choroid. A possible explanation for the significant negative correlation of FPT with age is insufficient nutrients and ischemia due to the accumulation of age-related deposits in the Bruch membrane and RPE, resulting in cone photoreceptor dysfunction. Histological sections have shown a decrease in cone photoreceptors at the parafoveal area in individuals above the age of 40 years (Panda-Jonas et al., 1995).

We found no relationship between cFPT with axial length, similar to other studies among myopes (Gella et al., 2011). Animal studies have shown that the photoreceptor and RPE layers are the least affected as compared with other retinal layers in eyes with induced myopia (Abbott et al., 2011). Human studies suggest that thinning occurs more in the middle to inner retinal layer with higher axial length (Wolsley et al., 2008). It appears that the photoreceptor layer is more adaptable to the effects of mechanical changes in high myopia.

The choroid is responsible for blood supply to the outer retina including the photoreceptor. However, we found no correlation between SFCT and FPT. It is still possible that the photoreceptor layer also receives blood supply from the central retinal artery. The thickness of the photoreceptor layer may be affected following any insult involving the inner retinal layer and central retinal artery. Further study to determine the exact blood supply of the photoreceptor should be performed to find out the reason for these unique features.

### Predictive Factors of Best Corrected Visual Acuity

We found BCVA to be positively correlated with age and axial length, with poorer BCVA associated with advancing age and longer axial length. The relationship of BCVA with age found in our study was in contrast with other reports which found a positive significant correlation of BCVA with age but no significant correlation with axial length (Nishida et al., 2012; Flores-Moreno et al., 2013).

A negative significant correlation was found between BCVA with both cFPT and cSFCT, conforming to other reports (Nishida et al., 2012; Flores-Moreno et al., 2013). However, a multiple linear regression showed that age, axial length, and cFPT were associated with poorer BCVA, whereas cSFCT is no longer associated with BCVA. Other authors have suggested theories to explain the association between reduced visual acuity with advancing age and increasing axial length (Curcio et al., 2000; Lam et al., 2007). As the axial length increased, pulsatile blood flow will decrease leading to cellular ischemia and a further reduction in cone density (Lam et al., 2007). Nutrient insufficiency has also been postulated to cause ischemia and reduction in cone density with advancing age (Curcio et al., 2000). The resultant reduction in cone density may attribute to reduced visual acuity.

Taking manual measurements may have its drawbacks and limits the reproducibility of the measurements taken in this study. We suggested a larger population-based study looking into normative values of choroidal and photoreceptor thickness in mild, moderate, and high myopia, using automated software to measure these thicknesses.

## Conclusion

Foveal photoreceptor thickness, age, and axial length are important factors for good visual acuity in highly myopic eyes. Thinner SFCT does not translate into poorer BCVA in these eyes.

## Data Availability Statement

The raw data supporting the conclusions of this article will be made available by the authors, without undue reservation.

## Ethics Statement

The studies involving human participants were reviewed and approved by the Research Governance Committee, UKM Medical Center, Cheras, Kuala Lumpur. The patients/participants provided their written informed consent to participate in this study.

## Author Contributions

MM, AA, M-LB, and ND: substantial contributions to the conception or design of the work and drafting the work or revising it critically for important intellectual content. RO, NR, AS, SK, ND, and AN: acquisition, analysis, or interpretation of data for the work. ND, SK, and M-LB: providing approval for publication of the content. MM, ND, and SK: agree to be accountable for all aspects of the work in ensuring that questions related to the accuracy or integrity of any part of the work are appropriately investigating and resolving. All authors contributed to the article and approved the submitted version.

## Conflict of Interest

The authors declare that the research was conducted in the absence of any commercial or financial relationships that could be construed as a potential conflict of interest.

## Publisher’s Note

All claims expressed in this article are solely those of the authors and do not necessarily represent those of their affiliated organizations, or those of the publisher, the editors and the reviewers. Any product that may be evaluated in this article, or claim that may be made by its manufacturer, is not guaranteed or endorsed by the publisher.
